# Exosomal miR-552-5p promotes tumorigenesis and disease progression via the PTEN/TOB1 axis in gastric cancer

**DOI:** 10.7150/jca.66903

**Published:** 2022-01-01

**Authors:** Lingyu Zhu, Suisui Zhang, Songda Chen, Huijie Wu, Mengjie Jiang, Aiqun Liu

**Affiliations:** 1 Department of Endoscopy Center, Guangxi Medical University Cancer Hospital, Nanning, Guangxi, 530021, China; 2 Department of Anesthesiology, The Second Affiliated Hospital of Chongqing Medical University, 400010, Chongqing, China.

**Keywords:** Exosomes, miR-552-5p, gastric cancer, PTEN, TOB1

## Abstract

**Purpose** Gastric cancer (GC) is associated with rapid disease progression and poor patient prognosis, highlighting the pressing need for new biomarkers to facilitate disease management. Exosomes are released by all cells and are ubiquitous in body fluids, thus giving them great potential as diagnostic biomarkers and therapeutic targets. MicroRNAs (miRNAs) can be transported by exosomes, and are a common target for regulation in cancer.

**Methods** Our screen of miRNAs in the Gene Expression Omnibus and The Cancer Genome Atlas databases identified miR-552-5p as the most overexpressed miRNA in GC, and we investigated its function and mechanism of action.

**Results** We detected high expression of miR-552-5p in GC tissues, plasma samples and cell lines. We found that miR-552-5p binds directly to the 3′-untranslated region of PTEN, and the resulting downregulation of PTEN in turn downregulates the tumor suppressor TOB1. Furthermore, experiments in cell culture and mice showed that miR-552-5p in exosomes is internalized by recipient cells, where it enhances proliferation, migration and the epithelial-mesenchymal transition, while suppressing the caspase-3 apoptotic pathway. These effects were reversed by inhibiting miR-552-5p.

**Conclusion** GC-derived exosomal miR-552-5p facilitates tumorigenesis by interfering with the PTEN/TOB1 axis, providing new potential therapeutic targets.

## Introduction

Gastric cancer (GC) is one of the most malignant tumors, with an estimated 1 million new diagnosed cases worldwide annually. Due to rapid disease progression and poor patient prognosis, GC is the third highest cause of cancer-related deaths 1. Thus, early detection, early diagnosis and early treatment are priorities to reduce morbidity and mortality of GC. Identifying effective biomarkers and exploring the potential disease mechanisms can provide new insights for developing GC therapies.

Exosomes may be a promising source of biomarkers for cancer detection and diagnosis as well as prediction of prognosis. Exosomes are extracellular vesicles of 30-150 nm in diameter that B cells, T cells, epithelial cells and tumor cells release into the extracellular environment via the endosomal vesicle pathway 2. Exosomes derived from tumors play a crucial role in intercellular communication by delivering cellular components such as proteins, messenger RNAs (mRNAs) and microRNAs (miRNAs) to neighboring and distant tissues 3. Exosomes abundant in the tumor microenvironment can accelerate tumor progression by regulating angiogenesis, modulating immunoreactivity, and mediating pre-metastatic niche formation 4-5. Recently, circulating miRNAs packaged in exosomes were shown to be transmitted between cells, affecting the tumor microenvironment, tumor cell infiltration and immunotherapy 6-7. A miRNA is a non-coding single-stranded RNA approximately 22 nucleotides long that can bind to the 3' untranslated region (UTR) of a target gene to inhibit its translation 8. Several studies have established that miRNAs stimulate tumor proliferation, metastasis, angiogenesis and drug resistance in multiple cancers including GC by regulating the expression of target genes 9-10.

The miR-552 is located on chromosome 1p34.3 and encodes a small non-coding RNA involved in post-transcriptional regulation. The two main members of this family are hsa-miR-552-5p and hsa-miR-552-3p, which are highly conserved 11. miR-552 is significantly overexpressed in several tumor types, where it drives cell proliferation, invasion, migration, and drug resistance, thereby accelerating tumorigenesis 12-14. Studies have initially explored miR-552 as a prognostic factor in GC 15. However, its role in the disease and the molecular mechanisms involved have not yet been clarified.

In the current study, we found that miR-552-5p was highly expressed in GC tissues and cells, indicating that miR-552-5p may be a potential prognostic factor. In particular, miR-552-5p promoted GC cell proliferation and migration, while inhibiting apoptosis. The miRNA was carried in exosomes, enabling its spread and cellular exchange. Exosomal miR-552-5p derived from a human gastric cancer cell line (SGC-7901) facilitated the malignant transformation of gastric epithelium (GES-1) and gastric adenocarcinoma (AGS) cells, and the effects were associated with inhibition of the synergy between the tumor suppressor “transducer of ERBB2.1” (TOB1) and PTEN. These findings highlight the potential of targeting this miRNA to treat GC.

## Material and methods

### Tissue and plasma specimens

The clinical samples were collected from June 2020 to January 2021. Gastric cancer tissue specimens and the corresponding adjacent noncancerous tissues were all from patients undergoing surgery in Guangxi Medical University Cancer Hospital. Normal blood samples were obtained from healthy volunteers. The tissue and blood samples were obtained from patients without receiving radiotherapy and chemotherapy before. All patients provided written informed consent, and the study was approved by the Cancer Institute of Guangxi Medical University and the hospital ethics committee. The specimens were immediately frozen in liquid nitrogen and stored at -80 °C.

### Cell culture

The cell lines GES1, SGC-7901, MGC-803 and AGS were purchased from the Chinese Academy of Sciences ATCC cell bank and cultured in RPMI 1640 medium (Gibco, USA) or Dulbecco's Modified Eagle's Medium (DMEM; Gibco, USA) containing 10% fetal bovine serum (FBS; Biological Industries, Israel) and 1% penicillin-streptomycin in a humidified incubator at 37 ℃ in an atmosphere of 5% CO_2_.

### Exosome extraction

The exosome-free FBS was obtained by centrifugation at 100,000 *g* at 4 °C for 16 h 16. SGC-7901 were cultured for 48 h in DMEM until about 80% confluence, then their medium was replaced with exosome-free conditioned medium. After incubation for 48 h, the culture medium was harvested, followed by centrifugation at 300 *g* at 4 °C for 10 min and at 2000 *g* at 4 °C for 20 min to remove dead cells and cell debris. Then the larger vesicles were removed by centrifugation at 10,000 *g* at 4 °C for 30 min. The supernatant was filtered through a 0.22-µm filter (Millipore, USA), and the exosomes were pelleted by centrifugation at 100,000 g at 4° C for 70 min. The pellet was diluted with PBS and centrifuged at 100,000 g at 4 °C for 70 min. Finally, the exosome pellets were resuspended in PBS and stored at -80 ℃.

### Transmission electron microscopy (TEM)

The resuspended exosomes were mixed thoroughly, after which 5 µL of exosomes were applied onto a formvar carbon-coated copper grid and allowed to adsorb for 5 min. The suspension was then negatively stained using 2% uranyl acetate solution, and the density and size of exosomes were determined under an electron microscope (Hitachi, Japan).

### RNA extraction and fluorescence-based quantitative PCR

Total RNA from tissue and cells was extracted using TRIzol reagent, and RNA from plasma samples was obtained using a miRNeasy serum/plasma kit according to the manufacturer's instructions (Qiagen, Germany). Exosomal RNA was purified using an exoRNeasy serum/plasma Midi Kit (Qiagen, Germany). Next, reverse transcription of mRNA or miRNA was carried out using the PrimeScript™ RT reagent Kit (TaKaRa, Japan) or Mir-X miRNA First-Strand Synthesis Kit (TaKaRa). Afterwards, qRT-PCR reactions were performed using the TBGreen® Premix Ex Taq™ II reagent (TaKaRa) with the following protocol: 95 ℃ pre-denaturation for 30 s, followed by 40 cycles of 95 ℃ denaturation for 5 s and 60 ℃ annealing for 30 s. U6 and β-actin were used as internal controls to normalize the expression levels of miRNA or mRNA, respectively. Relative expression was determined using the 2^-ΔΔCT^ method.

The following primers were used: has-miR-552-5p forward, 5'-ATTTAACCTTTTGCCTGTTGGAA-3'; TOB1 forward, 5'-CCCAGGTTTTTATGCCCATAAG-3'; TOB1 reverse, 5'-GTGGCAGTGGTAAAGGTTAAAG-3'; PTEN forward, 5'-GACCAGAGACAAAAAGGGAGTA-3'; PTEN reverse, 5'-ACAAACTGAGGATTGCAAGTTC-3'; U6 forward, 5'-GGAACGATACAGAGAAGATTAGC-3'; U6 reverse, 5'-TGGAACGCTTCACGAATTTGCG-3'; β-actin forward, 5'-GGCATCCTCACCCTGAAGTA-3'; β-actin reverse, 5'- GGGGTGTTGAAGGTCTCAAA-3'.

### Lentiviral transfection

Lentiviral plasmids were synthesized by Genechem (Shanghai, China) to overexpress miR-552-5p (hereafter: “Lv-miR-552-5p”) or silence it (“Lv-inhibitor”), to encode the corresponding negative controls “Lv-NC” and “Lv-ihNC”, and to overexpress PTEN (“oe-PTEN”) and the corresponding negative control (“oe-NC”). Cells were seeded in a 6-well plate (5x10^4^ cells/mL), transfected with lentiviral plasmids and cultured for 3 days, followed by selection in 1.5 μg/mL puromycin. The expression levels of miR-552-5p and PTEN were verified by quantitative PCR.

### Cell counting kit‑8 (CCK‑8) assay

Cells that had been transfected with plasmids or treated with exosomes were harvested, centrifuged, and inoculated into a 96-well plate at a density of 2 × 10^3^ cells/well (100 µL) containing DMEM or RPMI 1640 supplemented with 10% FBS. At 24, 48, 72 and 96 h, 10 μL of prepared CCK-8 solution was added to each well, and the plate was incubated for 2 h at 37 ℃ in an atmosphere of 5% CO_2_. Optical density at 450 nm (OD_450_) was measured using an automated microplate reader.

### Colony formation assay

Cells were harvested and seeded into a 6-well plate at 1×10^3^ cells/well, then incubated for 9-12 days. After washing with PBS, the cells were fixed in 4% paraformaldehyde for 30 min, and then dyed with 0.1% crystal violet for 30 min at room temperature.

### Assay with 5-ethynyl-2'-deoxyuridine (EdU)

Cells were harvested and seeded in 96-well plates at 2×10^4^ cells/well. In accordance with the manufacturer's protocol (RiboBio), cells in logarithmic growth phase were incubated with EdU solution for 2 h, and fixed with methanol for 30 min at room temperature. Cells were washed with PBS, permeabilized with 0.1% Triton X-100 in PBS for 10 min, then incubated with 1x Apollo reaction solution in the dark for 30 min. For DNA staining, the cells were incubated with Hoechst 33342 for 30 min. The cells with red fluorescent nuclear staining were counted as positive.

### Cell migration assay

A transwell assay was performed to assess the migration ability of cells. The treated cells were resuspended in serum-free DMEM or RPMI 1640 at 1×10^5^ cells/mL, and 200 µL was inoculated into the upper chamber, while 500 µL DMEM or RPMI 1640 with 10% FBS was added into the lower chamber. Cultures were incubated in a humidified incubator at 37 ℃ in an atmosphere of 5% CO_2_ for 48 h. The cells on the topside of the filter were removed with a cotton swab. The membrane was fixed with 4% paraformaldehyde for 30 min, then stained with 0.1% crystal violet for 30 min. After drying, the number of migrated cells was quantified under a microscope.

### Apoptosis assay

Cell apoptosis was detected using the Annexin V-APC/7-AAD Apoptosis Detection Kit (Hangzhou Multisciences Biotech, Hangzhou, China) according to the manufacturer's instructions. Cells were harvested using EDTA-free trypsin, centrifuged and washed with ice-cold PBS. The cell suspension (5×10^6^ cells /mL) was mixed with 500 µL 1× binding buffer, 5 μL Annexin V-APC and 10 μL 7-Aminoactinomycin D (7-AAD). After incubation in the dark at room temperature for 15 min, the stained cells were analyzed by BD FACS Calibur flow cytometry. The proportion of apoptotic cells was calculated by adding together the proportions of cells in early apoptosis (Annexin V-APC+, 7-AAD-) and late apoptosis (Annexin V-APC+, 7-AAD+).

### Western blot analysis

Tissues, cells or exosomes were lysed in radioimmunoprecipitation lysis buffer (RIPA) containing PMSF (100:1 dilution), and total protein concentration was determined using a BCA protein quantification kit. The protein samples were separated through 10% SDS-containing polyacrylamide gel (SDS-PAGE), then transferred onto 0.22-μm polyvinylidene difluoride (PVDF) membranes. Next, the membranes were blocked in TBST containing 5% skimmed milk for 1.5 h. After washing with PBS three times, PVDF membranes were incubated at 4 °C overnight with antibodies against the following proteins: TSG101 (Immunoway, USA), CD9 (Immunoway), ALIX (Immunoway), PTEN (Immunoway), TOB1 (Immunoway), BCL-2 (Immunoway), BAX (Immunoway), cleaved-caspase 3 (CST, USA), E-cadherin (CST), N-cadherin (CST), vimentin (CST), or β-Actin (CST). The membranes were washed three times with PBS, then incubated with horseradish peroxidase-conjugated anti-rabbit IgG secondary antibody for 1 h at room temperature. The protein bands were visualized by enhanced chemiluminescence, and the relative expression of target proteins was normalized to β-Actin.

### Luciferase reporter assay

The binding sites of miR-552-5p in the 3'-UTRs of the TOB1 and PTEN mRNAs were predicted by TargetScan (http://www.targetscan.org/vert_72/). The wild-type 3'-UTR of TOB1 or PTEN, or mutated 3'-UTRs lacking the miR-552-5p binding sites, were inserted downstream of the luciferase coding sequence in the pMIR-REPORT plasmid, and then co-transfected into 293T cell along with an miR-552-5p-mimic or NC-mimic using Lipofectamine 2000 (Invitrogen). After 48 h, luciferase activity was assayed utilizing a Dual-Luciferase Reporter Assay System (no. E1960, Promega, USA). The relative luciferase activity was reported as the ratio of firefly luciferase to *Renilla* luciferase activity.

### Co-immunoprecipitation

Total protein from oe-PTEN or oe-NC cells (500 µL) was mixed with 40 µL of Anti-Flag M2 Affinity Gel, washed twice with 1 mL TBS, and incubated at 4 ℃ overnight. After washing with TBS, the supernatant was harvested and denatured with 5x protein loading buffer (Beyotime, Shanghai) at 100 ℃. The expression of Flag and TOB1 was subsequently measured using western blot analysis.

### Exosome labeling

Exosomes were purified as described in section 2.3 and incubated with 5 μM Dil dye at room temperature in the dark for 10 min. Reactions were terminated by addition of an equal amount of 1% BSA. Exosomes were purified using an exosomal spin column with a molecular weight cut-off of 3 kDa (Thermo Fisher Scientific, USA). Then, AGS cells and GES-1cells were incubated for 12 h at 37 °C with Dil-labeled exosomes in confocal dishes in a humidified atmosphere of 5% CO_2_. The cells were subsequently fixed in methanol for 15 min, then permeabilized and stained with Hochest 33342 for 15 min. After washing with PBS, the uptake of exosomes was examined under a confocal microscope.

### Animal studies

Four-week-old BALB/c female nude mice were randomly divided into four groups (n=5 per group). Lv-miR-552-5p, Lv-NC, Lv-ihNC and Lv-inhibitor cultures were harvested, centrifuged, resuspended in 200 µL PBS (5x10^6^ cells/ml), then subcutaneously injected into the inguinal region into the mice. The size of tumors was measured using a Vernier caliper every three days. In parallel, another set of mice were assigned to five analogous exosome-treated groups: PBS, Exo-Lv-miR-552-5p, Exo-Lv-NC, Exo-Lv-ihNC and Exo-Lv-inhibitor. At 18 days later, the xenografts were harvested and frozen in liquid nitrogen or fixed in 4% paraformaldehyde for subsequent analysis. The tumor volume was calculated using the following formula: V = (width^2^ × length) / 2.

The cells were resuspended in PBS at 2×10^6^ cells/mL, then injected into the left or right caudal vein of female BALB/c nude mice. After 8 weeks, the mice were sacrificed by cervical dislocation, and the lung tissues were collected and immersed in 10% neutral formalin for hematoxylin staining.

### Immunohistochemistry and hematoxylin staining

Tumor xenografts in each group were fixed with 4% paraformaldehyde, embedded in paraffin and sliced. The sections were incubated at 60 ℃ for 2 h, dewaxed in xylene and dehydrated through an ethanol gradient. After antigen repair, the sections were incubated successively with catalase blocking solution, normal non-immune animal serum, then with anti-Ki67 antibody (Santa Cruz Biotechnology, USA) at 4 °C overnight. Sections were rewarmed for 30 min, then incubated with secondary antibody for 10 min at room temperature. Finally, the slides were stained with freshly prepared 3,3′-diaminobenzidine (DAB) and hematoxylin, then analyzed using light microscopy (Olympus BX43, Japan).

The sections of lung metastases were treated sequentially with xylene and an ethanol gradient, then immersed in hematoxylin solution, differentiated with hydrochloric alcohol and stained with eosin. Two experienced pathologists who were unaware of the experimental conditions evaluated tumor metastasis.

### Statistical analysis

SPSS 20 was used for statistical analysis, and the results were presented as mean ± SD. Independent sample t test and one-way analysis of variance (ANOVA) were used to examine the statistical significance between two groups and among multiple groups. Statistical significance was defined as *p < 0.05, **p < 0.01, and ***p < 0.001.

## Results

### The miR-552-5p is highly expressed in GC

**Figure 1:**
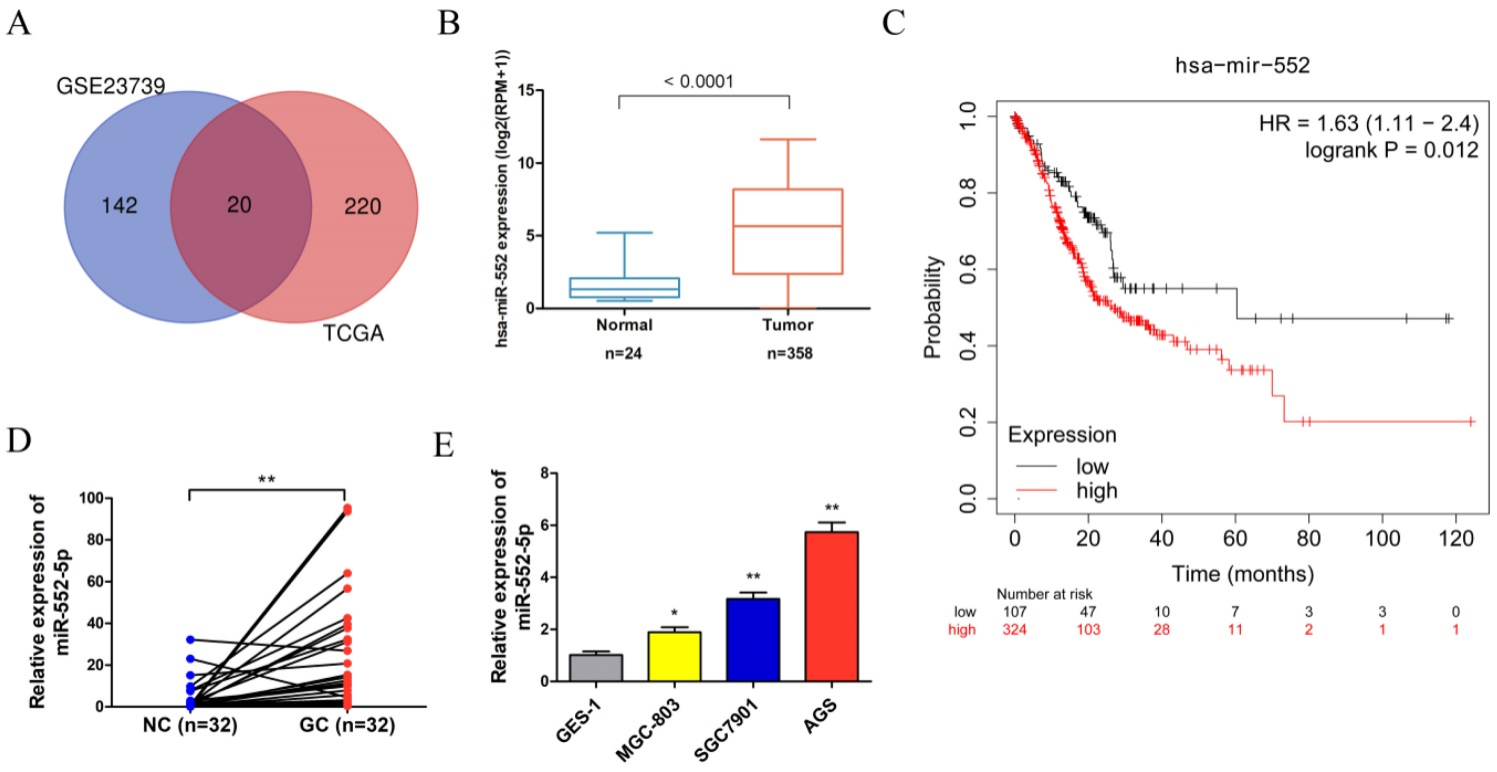
** The expression of miR-552-5p is upregulated in GC.** A. Venn diagram showing that 20 miRNAs are significant differential expression in both the TCGA and GSE23739 databases. B. The expression of miR-552 is significantly upregulated in GC in the TCGA database. C. Kaplan-Meier survival analysis revealing that high expression of miR-552 is correlated with poor prognosis. D. qRT-PCR analysis of miR-552-5p expression in GC tissues and paired adjacent noncancerous (NC) tissues (n=32). E. The expression of miR-552-5p in MGC-803, SGC-7901, AGS and GES-1 cells measured by qRT-PCR. *p < 0.05; **p < 0.01; ***p < 0.001.

Analysis of the Gene Expression Omnibus (GEO) database microarray data (GES23739) (https://www.ncbi.nlm.nih.gov/geo/) revealed that 162 miRNAs displayed significant expression differences using the limma package in the R language between 40 GC tissues and 40 adjacent non-cancerous (NC) tissues, based on cut-off criteria of |log2(fold change)| > 1.5 and adjusted P < 0.05. The project TCGA_STAD were downloaded from the GDC Data Portal (https://portal.gdc.cancer.gov/) using R package TCGA biolinks, revealing 240 miRNAs with significant expression differences based on cut-off criteria of |log2(fold change)| > 1 and adjusted P < 0.05. After integrating the analysis of GEO and TCGA data, 20 miRNAs were found in the intersection of the two data sets. The miR-552 was selected for further analysis because it was significantly differentially expressed and directly related to prognosis (Figure 1A). The miR-552 expression level was significantly higher in GC tissues than in adjacent control tissues in the TCGA database (Figure 1B). The Kaplan-Meier plot indicated that the survival rate of GC patients with high miR-552 expression was consistently lower than that of patients with low expression (Figure 1C). We verified the increased miR-552-5p level in paired GC tissues (n=32) using qRT-PCR, which corroborated our TCGA analysis (Figure 1D). Table 1 showed that the level of miR-552-5p was statistically related to tumor invasion depth, lymph node metastasis and TNM stages in GC patients. We next measured the expression of miR-552-5p in GC cells (MGC-803, SGC-7901, AGS) and normal non-cancerous gastric mucosal cells (GES-1) using qRT-PCR. AGS and SGC-7901 cells had significantly higher miR-552-5p expression than GES-1 cells (Figure 1E).

### The miR-552-5p promotes GC cell proliferation and migration and inhibits apoptosis

**Figure 2:**
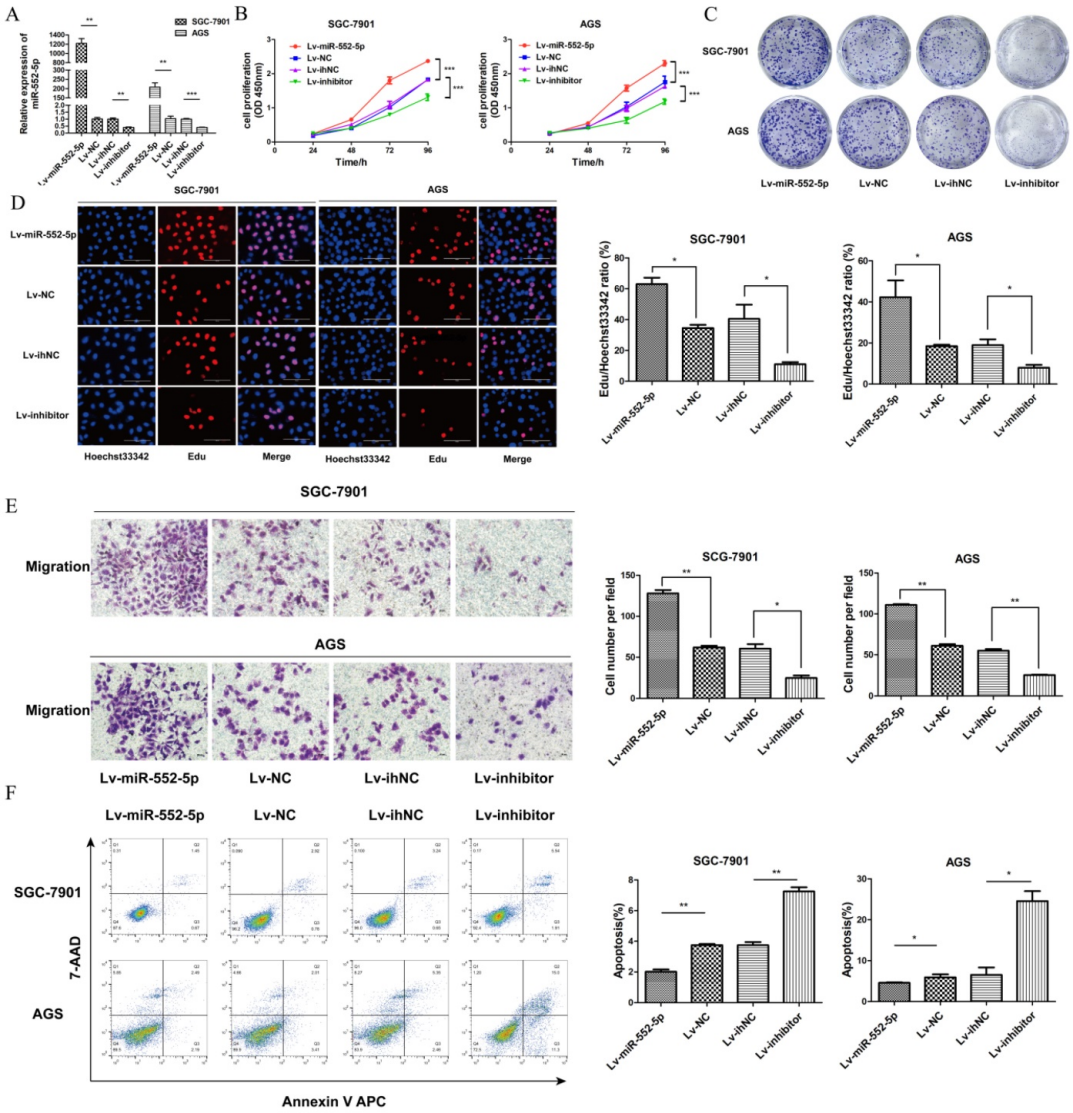
** The miR-552-5p mediates GC cell proliferation, migration and apoptosis *in vitro*.** A. Levels of miR-552-5p, as determined by qRT-PCR, in cells transfected with one of four lentiviral vectors: upregulating miR-552-5p (Lv-miR-552-5p) or silencing it (Lv-inhibitor), and the corresponding negative controls (Lv-NC and Lv-ihNC). Assays based on cell counting (CCK-8) (B), colony formation (C), and EdU incorporation (D) by transfected SGC-7901 and AGS cells. Scale bar, 100 µm. E. Cell migration, as measured by a transwell migration assay. F. Flow cytometry analysis showing apoptosis levels in transfected cells. The apoptotic cell ratio (%) is presented in a histogram. *p < 0.05; **p < 0.01; ***p < 0.001.

To investigate the potential role of miR-552-5p in GC, we constructed lentiviral vectors upregulating miR-552-5p (Lv-miR-552-5p) or silencing it (Lv-inhibitor), along with the corresponding negative controls (Lv-NC and Lv-ihNC). These constructs were transfected into SGC-7901 and AGS cells, leading to significant up- or down-regulation of miR-552-5p expression, as confirmed with qRT-PCR (Figure 2A). Upregulation of miR-552-5p (Lv-miR-552-5p) increased the proliferation of SGC-7901 and AGS cells, based on the CCK-8 (Figure 2B), colony formation (Figure 2C) and EdU assays (Figure 2D), while inhibition of miR-552-5p (Lv-inhibitor) exerted the opposite effects.

In addition, upregulation of miR-552-5p significantly promoted the migration of GC cells (Figure 2E) and it inhibited apoptosis in SGC-7901 and AGS cells (Figure 2F). Transfection with Lv-inhibitor exerted the opposite effects.

### The miR-552-5p directly targets PTEN and thereby downregulates TOB1

**Figure 3:**
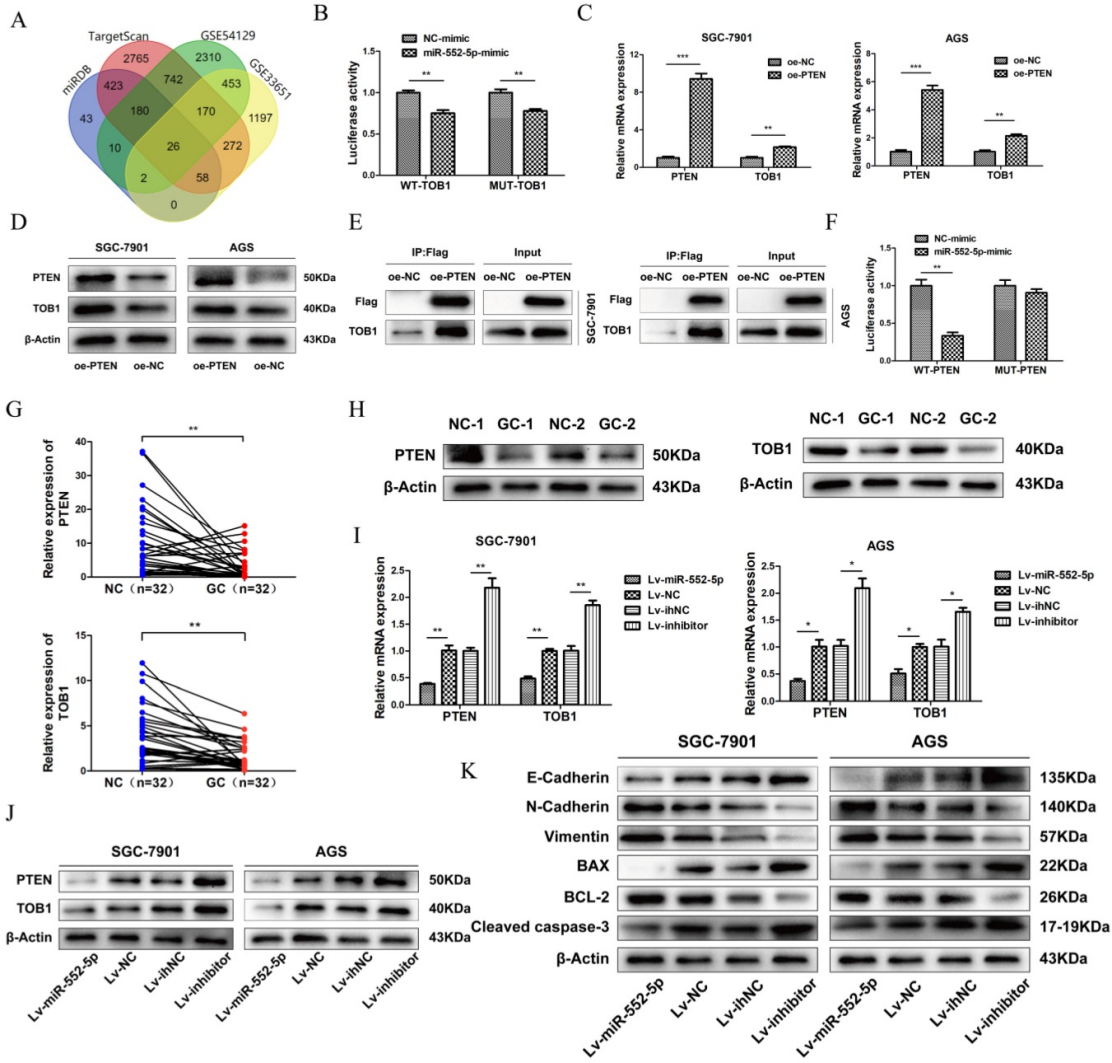
** The miR-552-5p directly targets PTEN and indirectly downregulates TOB1.** A. The miR-552-5p target genes predicted by miRDB, TargetScan, GSE54129 and GSE33651. B. The absence of binding between miR-552-5p and TOB1 illustrated by the relative levels of luciferase in 293T transfected with wild-type (WT) or mutant TOB1 3′-UTR and miR-552-5p mimics. qRT-PCR (C) and western blot (D) analysis verifying the levels of PTEN and TOB1 in SGC-7901 and AGS cells transfected with lentiviral vectors targeting PTEN or controls. E. Co-immunoprecipitation analysis of the interaction between TOB1 and FLAG-PTEN. F. Dual luciferase assay verifying the predicted binding site of miR-552-5p in the PTEN 3'-UTR. G. qRT-PCR analysis of PTEN and TOB1 mRNA expression in GC tissues and paired adjacent noncancerous (NC) tissues (n = 32). H. PTEN and TOB1 protein expression were analyzed in gastric tumor tissues and paired adjacent NC tissues using western blotting. qRT-PCR (I) and western blotting (G) were used to assess TOB1/PTEN expression in AGS and SGC-7901 cells transfected with Lv-miR-552-5p, Lv-NC, Lv-inhibitor or Lv-ihNC. K. Changes in miR-552-5p expression altered the expression of apoptosis-related proteins (cleaved caspase-3, Bax, Bcl-2) and EMT-related proteins (E-cadherin, N-cadherin and vimentin), as determined by western blotting analysis. β-actin was used as a loading control. *p < 0.05; **p < 0.01; ***p < 0.001.

Analysis of TargetScan, miRDB and GEO databases (GSE54129, GSE33651) revealed 26 differentially expressed mRNAs, with 3' UTR of TOB1 predicted as the most likely binding target (Figure 3A). TOB1 is a member of a family of anti-proliferative factors that act as tumor suppressors. We set up dual-luciferase reporter gene assays to determine the interaction between miR-552-5p and the TOB1 3'-UTR. While miR-552-5p affected the expression of luciferase containing the TOB1 3'-UTR, this regulatory ability did not significantly change after mutating the putative binding sites in the 3'-UTR. This suggests that miR-552-5p may not bind directly to the TOB1 mRNA (Figure 3B). A recent study showed PTEN to interact with TOB1 17, which we verified by qRT-PCR (Figure 3C), western blotting (Figure 3D) and co-immunoprecipitation (Figure 3E). A binding site of miR-552-5p on the PTEN 3' UTR was predicted by TargetScan, and this potential interaction was examined by co-transfecting miR-552-5p or NC mimics together with luciferase reporter plasmids where the luciferase gene carried the wild-type (WT) or mutant PTEN 3′-UTR. Luciferase activity decreased when miR-552-5p mimics were co-transfected with wild-type PTEN 3′-UTR, and this decrease was abolished when the mutated 3'-UTR was used (Figure 3F). These results indicate that miR-552-5p may bind directly to the PTEN 3'-UTR, and that the resulting downregulation of PTEN may also downregulate TOB1.

In support of this hypothesis, qRT-PCR (Figure 3G) and western blotting (Figure 3H) clearly revealed that both PTEN and TOB1 were expressed at higher levels in control tissues than in GC tissues. As predicted, overexpression of miR-552-5p decreased the mRNA and protein levels of PTEN and TOB1, and this effect was reversed by downregulation of miR-552-5p (Figure 3I-J).

The epithelial-mesenchymal transition (EMT) refers to the cellular transition from epithelial to mesenchymal phenotypes, which is associated with early tumorigenesis and progression, intravascular invasion, and metastasis and therapy resistance 18. The decrease of epithelial cell surface markers and the increase of mesenchymal markers can act as indicators of cancer progression during the EMT 19. Therefore we assessed the expression of apoptosis-related proteins (cleaved caspase-3, Bax, Bcl-2) and EMT-related proteins (E-cadherin, N-cadherin and vimentin) by western blotting. Upregulation of miR-552-5p led to an increase in N-cadherin and vimentin and a reduction in E-cadherin, while the expression of cleaved caspase-3 and Bax were inhibited, and the expression of Bcl-2 was increased (Figure 3K). Lv-inhibitor led to the opposite effects.

### The miR-552-5p accelerates the EMT in GC cells by inhibiting the PTEN/TOB1 axis

**Figure 4:**
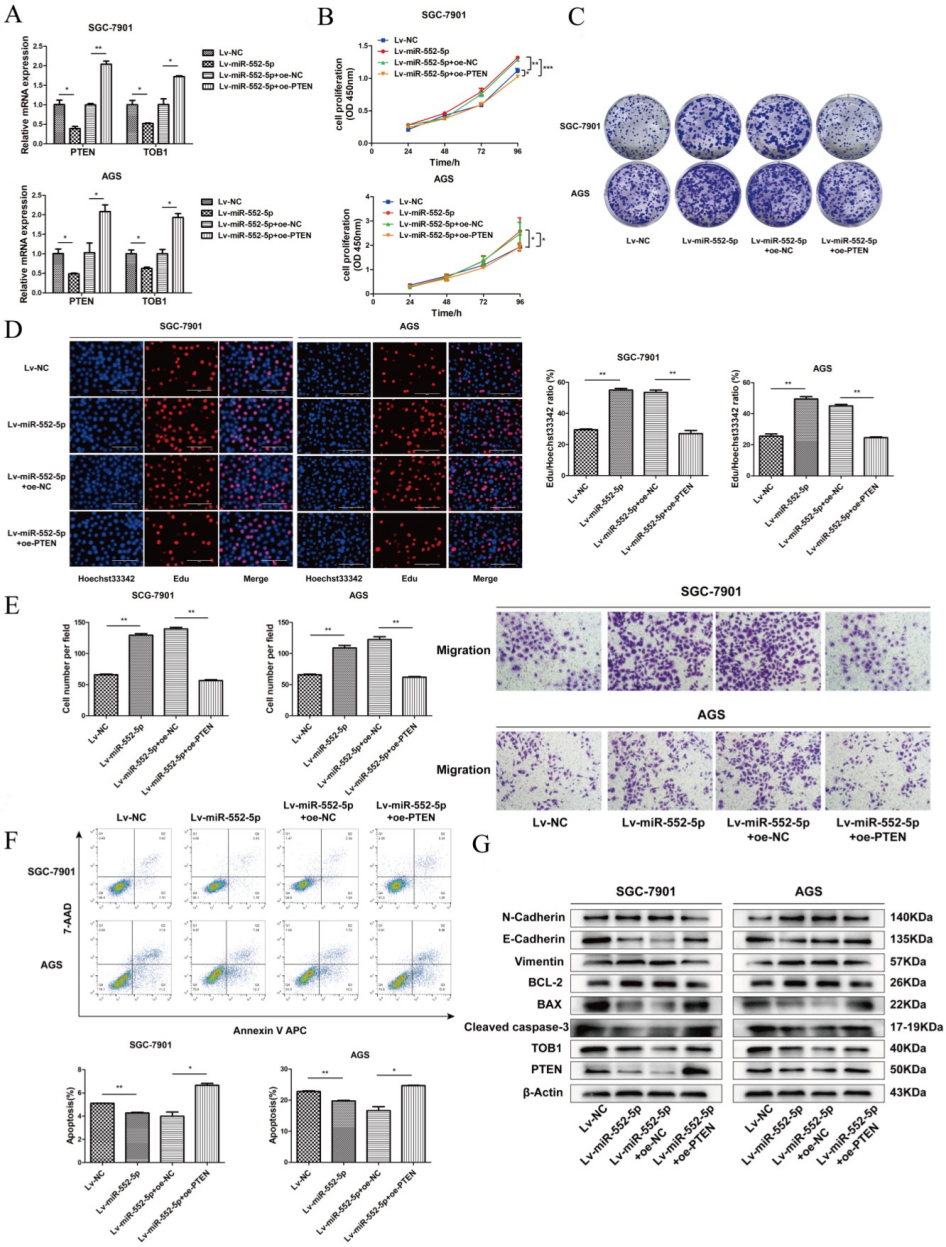
** The miR-552-5p accelerates EMT progression of GC cells by regulating PTEN/TOB1 expression.** A. The mRNA levels of PTEN and TOB1 examined by qRT-PCR after transfection with Lv-miR-552-5p or Lv-NC, in combination with oe-PTEN or oe-NC. Assays of CCK-8 (B), colony formation (C) and EdU incorporation (D) illustrate the proliferation of SGC-7901 and AGS cells in each group. Scale bar, 100 µm. E. Cell migration ability assessed by a transwell migration assay. F. Flow cytometry analysis of cell apoptosis. The apoptotic cell ratio (%) is presented in a histogram. G. Levels of apoptosis- and EMT-related proteins evaluated by western blotting. β-actin was used as a loading control. *p < 0.05; **p < 0.01; ***p < 0.001.

To verify that miR-552-5p can inhibit PTEN/TOB1 expression to accelerate tumor progression, plasmids to overexpress PTEN (oe-PTEN) or a negative control (oe-NC) were stably transfected into SGC-7901 and AGS cells expressing Lv-miR-552-5p. Levels of mRNAs encoding PTEN and TOB1 were substantially higher in cells overexpressing PTEN (Figure 4A). Such cells also showed significantly lower proliferation based on CCK-8 (Figure 4B), colony formation (Figure 4C), and EdU assays (Figure 4D). In SGC-7901 and AGS cells overexpressing miR-552-5p, further up-regulation of PTEN levels reversed the promotion of migration (Figure 4E) and inhibition of apoptosis by miR-552-5p (Figure 4F). PTEN overexpression in such cells also downregulated Bcl-2, N-cadherin and vimentin, while upregulating PTEN, TOB1, cleaved caspase-3, Bax and E-cadherin (Figure 4G).

### The miR-552-5p stimulates tumorigenesis and metastasis *in vivo*

**Figure 5:**
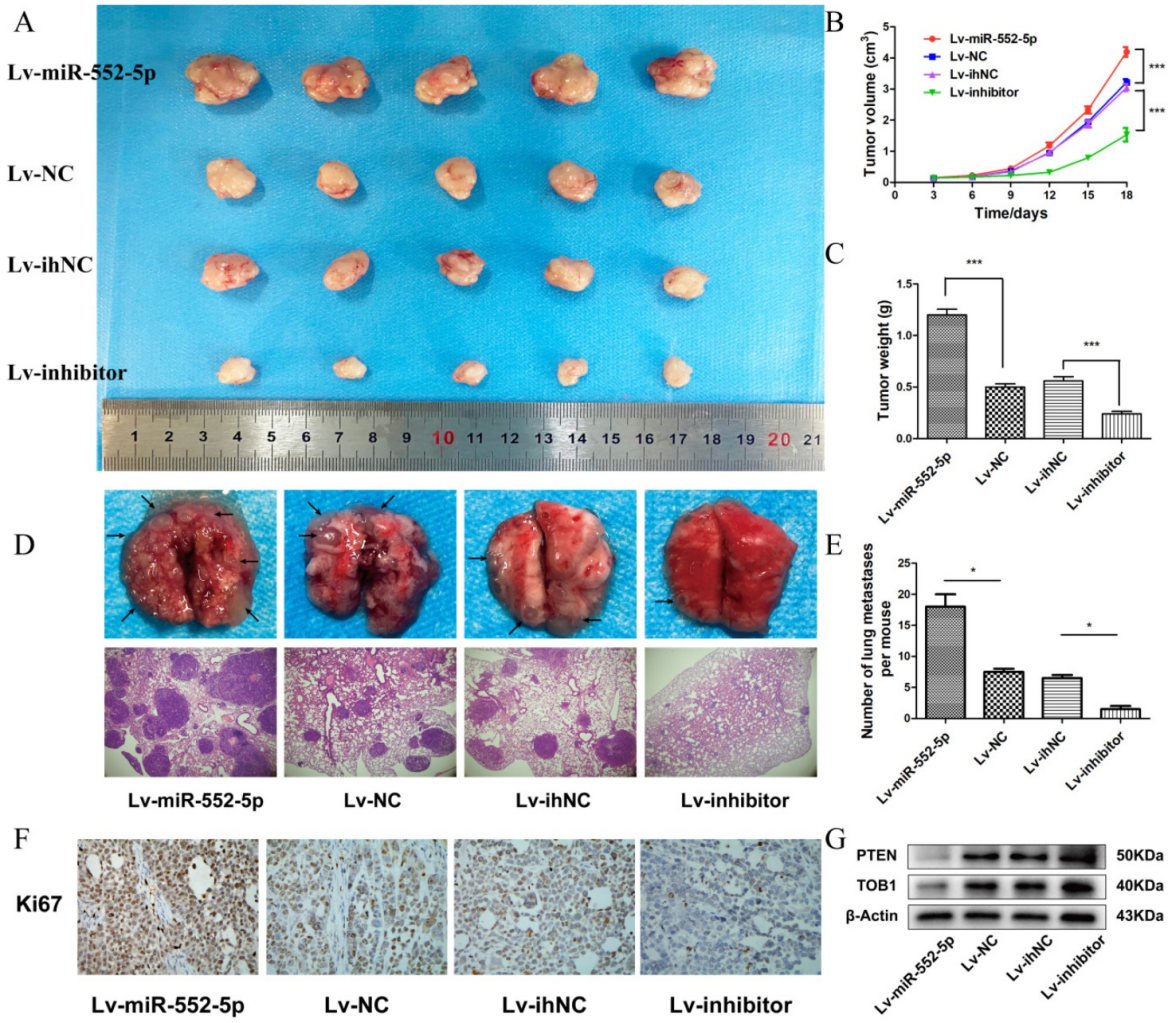
** The miR-552-5p stimulates tumorigenesis and metastasis through the PTEN/TOB1 axis in a GC xenograft mouse model.** A. Harvested xenografts from mice injected with SGC-7901 cells transfected with the indicated lentiviral constructs. B. Tumor growth curve in the different animal groups. C. Tumor weights in the different animal groups. D. Representative image of lung metastasis in each mouse group (magnification ×40). E. The number of lung metastasis nodules in each mouse group. F. Immunohistochemical analysis of xenograft tissues performed using an anti-Ki67 antibody (magnification ×200). G. The expression of PTEN and TOB1 in xenograft tumors detected by western blotting. β-actin was used as a loading control. *p < 0.05; **p < 0.01; ***p < 0.001.

We next investigated whether miR-552-5p exerts pro-tumorigenic effects *in vivo*. SGC-7901 cells transfected with Lv-miR-552-5p, Lv-NC, Lv-inhibitor or Lv-ihNC were subcutaneously injected into BALB/c nude mice, and the size of the xenografts were measured every three days with a caliper. After 18 days, the mice were sacrificed. The harvested xenografts are shown in Figure 5A, which revealed that increased miR-552-5p expression contributed to tumor proliferation, whereas reduced miR-552-5p expression suppressed tumorigenesis. In addition, tumor volume (Figure 5B) and weight (Figure 5C) were significantly higher in miR-552-5p animals, while loss of miR-552-5p was accompanied by a decrease in tumor volume and weight. The expression of miR-552-5p further stimulated distant metastasis of SGC-7901 cells, leading to more extensive lung metastases than in the control groups (Figure 5D-E). These effects were reversed by Lv-inhibitor.

Immunohistochemical analysis revealed a higher percentage of Ki67-positive tumor cells in Lv-miR-552-5p animals than in controls, and a lower percentage in Lv-inhibitor animals than in controls (Figure 5F). Western blotting indicated a reduction in PTEN and TOB1 levels in Lv-miR-552-5p animals, and an increase in their levels in Lv-inhibitor animals (Figure 5G).

### The miR-552-5p is upregulated in GC-derived exosomes

**Figure 6:**
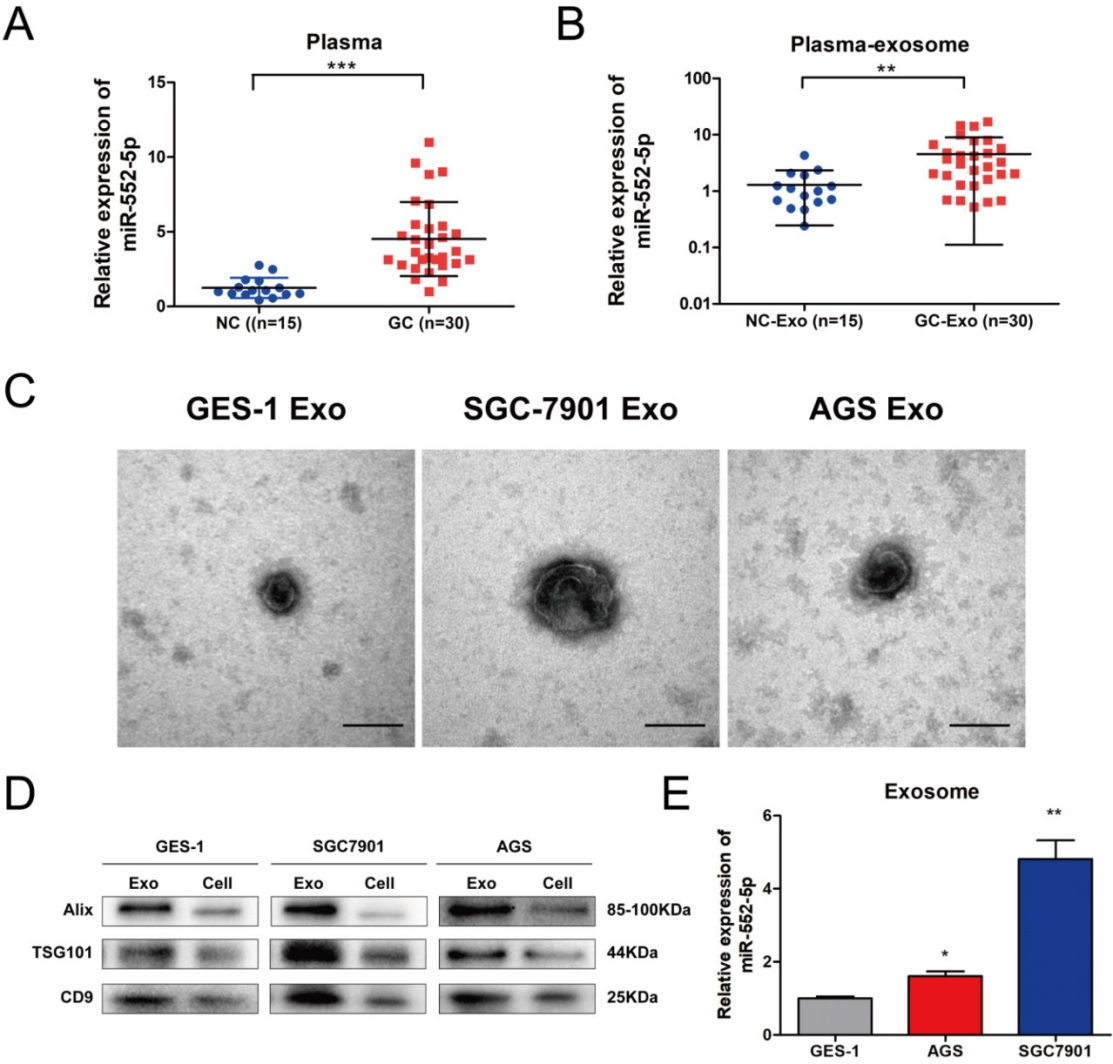
** The miR-552-5p is upregulated in exosomes derived from GC plasma and cells.** A. Relative expression levels of miR-552-5p in the plasma from GC patients (n=30) and healthy volunteers (n=15), analyzed by qRT-PCR. B. Relative levels of miR-552-5p in plasma exosomes from GC patients (n=30) and healthy volunteers (n=15), as analyzed by qRT-PCR. C. Representative electron micrographs of exosomes isolated from the medium of cultures of SGC-7901, BGC-823 and GES-1 cells. Scale bar, 100 nm. D. Western blotting analysis of the levels of exosome markers (TSG101, Alix and CD9). E. Relative expression of Exo-miR-552-5p in GES-1, SGC-7901 and AGS cells. *p < 0.05; **p < 0.01; ***p < 0.001.

Exosomes participate in signal transduction between adjacent and distant cells, and can facilitate tumor metastasis 20. Among them, exosomal miRNAs can serve as stable and prominent biomarkers of GC metastasis 21-22. Therefore, we compared the levels of miR-552-5p in plasma from GC patients (n=30) and healthy volunteers (n=15). Levels were significantly higher in patient plasma (Figure 6A) as well as in exosomes purified from patient plasma (Figure 6B). Clinical correlation analysis showed that the level of the plasma exosomal miR-552-5p had statistical significance with tumor invasion depth, lymph node metastasis and TNM stages in GC patients (Table 2). We next cultured GES-1, SGC-7901 and AGS cells in exosome-free conditioned medium and purified exosomes from the resulting culture medium, which appeared as approximately spherical vesicles when viewed by electron transmission microscopy (Figure 6C). Western blotting analysis confirmed the expression of exosome markers (including ALIX, TSG101 and CD9) (Figure 6D) and showed that levels of miR-552-5p in exosomes from SGC-7901 cells were remarkably higher than those in exosomes from the other two cell types (Figure 6E). This indicates that exosomes function as a protective carrier of miR-552-5p.

### Exosomal miR-552-5p derived from GC cells enhances tumor progression of recipient cells

**Figure 7:**
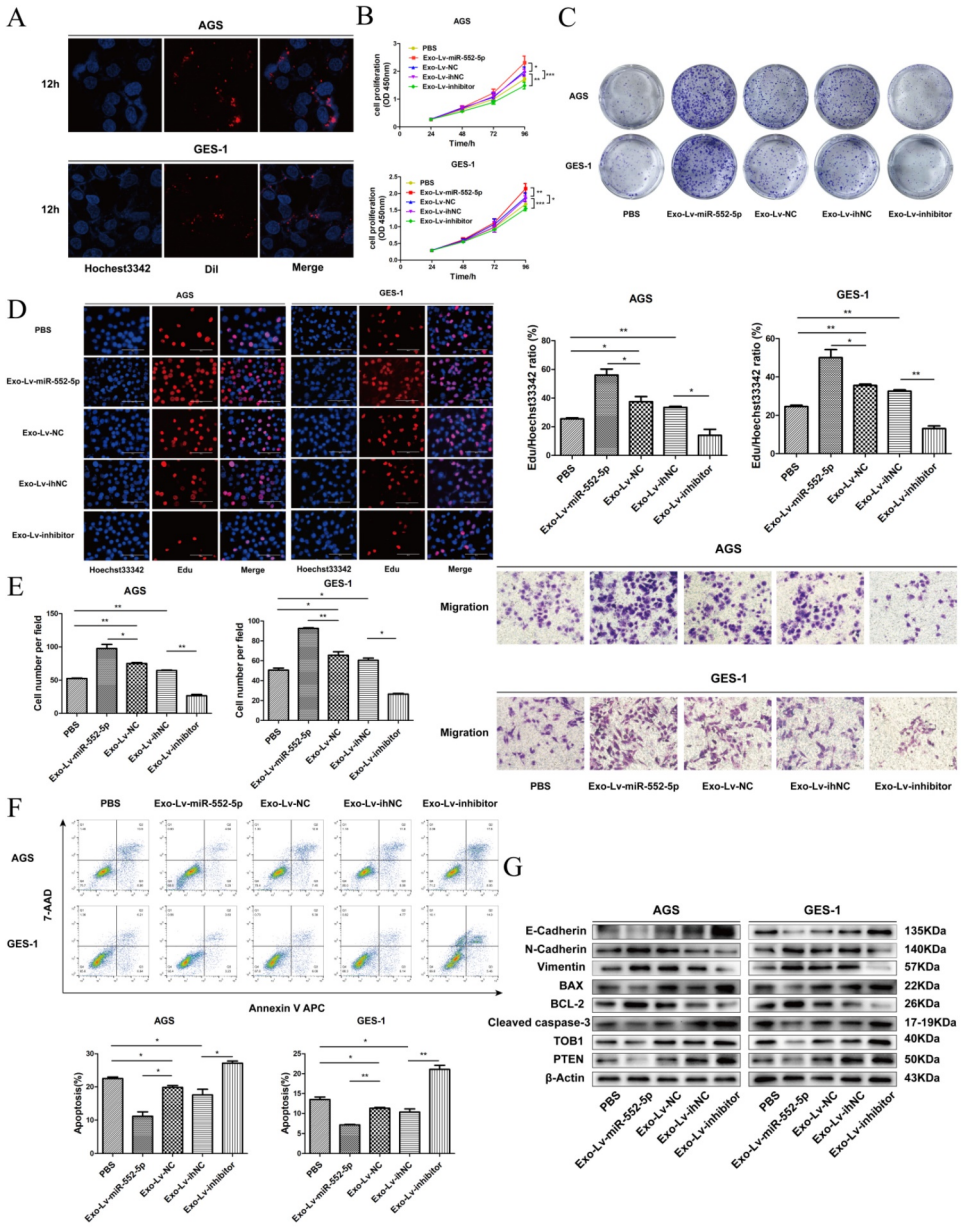
** Exosomal transfer of miR-552-5p enhances malignant transformation of recipient cells.** A. Cellular uptake of Dil-labelled exosomes (red) in GES-1 and AGS cells observed by confocal microscopy. Representative images were captured after 12 h of incubation with exosomes (magnification ×1000). The proliferation of GES-1 and AGS cells after exosome treatment was assessed using assays of CCK-8 (B), colony formation (C) and EdU incorporation (D). E. The migration of AGS and GES-1 cells was assessed after exosome uptake. F. Representative flow cytometric analysis of apoptosis of AGS and GES-1 cells treated with exosomes. G. Expression of PTEN, TOB1, BAX, BCL-2, cleaved caspase-3, E-cadherin, N-cadherin and vimentin in AGS and GES-1 cells after addition of exosomes, analyzed by Western blotting. β-actin was used as a loading control. *p < 0.05; **p < 0.01; ***p < 0.001.

To further observe the miR-552-5p transport and uptake process, exosomes from SGC-7901 cells transfected with different lentiviral vectors were labeled with Dil red dye and incubated with AGS and GES-1 cells, then stained with Hoechst 33342 blue dye for nuclear localization. Figure 7A shows the high intracellular uptake of exosomes in recipient cells. Next, we explored proliferation, migration, and apoptosis in exosome-treated (exo) recipient cells. The numbers and growth rates of cells co-cultured with exo-Lv-miR-552-5p were significantly higher than in control cells, as assessed by CCK-8 (Figure 7B), colony formation (Figure 7C) and EdU assays (Figure 7D), whereas the opposite trend was observed in exo-Lv-inhibitor cells. Moreover, exo-Lv-miR-552-5p also promoted migration of the recipient cells, while addition of exo-Lv-inhibitor clearly inhibited cell migration (Figure 7E). The numbers of apoptotic AGS and GES-1 cells decreased with exo-Lv-miR-552-5p treatment and, conversely, increased with exo-Lv-inhibitor treatment (Figure 7F). Treatment with exosomes carrying Lv-miR-552-5p decreased PTEN and TOB1 expression, which was accompanied by higher expression of Bcl-2, N-cadherin and vimentin and lower expression of cleaved caspase-3, Bax and E-cadherin. The opposite effects were observed after treatment with exosomes carring Lv-inhibitor (Figure 7G). These results collectively suggest that miR-552-5p inhibits the PTEN-TOB1 axis and caspase-3 apoptosis signaling, promoting the EMT.

### The miR-552-5p is delivered via exosomes to accelerate tumor growth by regulating the PTEN/TOB1 axis *in vivo*

**Figure 8:**
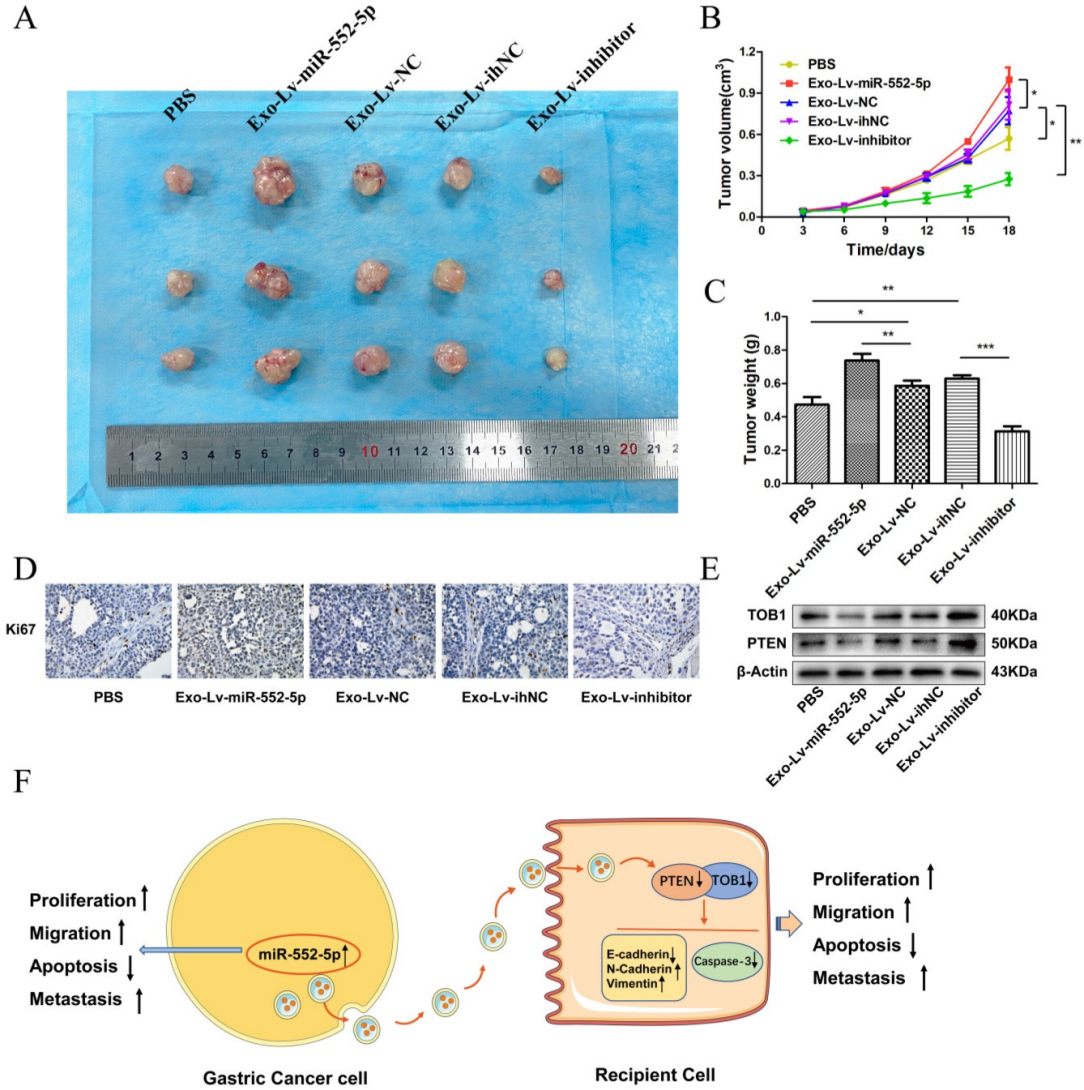
** Exo-miR-552-5p accelerates tumor growth *in vivo*.** A. Xenograft tumors excised from groups treated with different exosomes and controls. B. Tumor growth curve of xenografts after exosome treatment. C. Tumor weight in each mouse group. D. Immunohistochemical analysis of Ki67 expression in the xenografts (magnification ×200). E. Western blot analysis of PTEN and TOB1 protein expression in the different treatment groups. F. Schematic diagram of how GC cell-derived exosomes containing miR-552-5p may promote tumorigenesis and progression in recipient cells. Exo-miR-552-5p inhibits the PTEN/TOB1 axis to facilitate recipient cell proliferation, migration, metastasis, and EMT, while also inhibiting caspase-3 signaling and apoptosis. *p < 0.05; **p < 0.01; ***p < 0.001.

To further explore the effects of exosomal miR-552-5p delivery *in vivo*, we subcutaneously injected nude mice with AGS treated with exosomes carrying Lv-miR-552-5p, Lv-NC, Lv-inhibitor or Lv-ihNC. Control animals were injected AGS treated with PBS. Tumors were collected for subsequent immunohistochemistry and western blot analysis. Exo-miR-552-5p treatment led to larger tumors (Figure 8A) and stimulated tumor proliferation, based on the tumor growth curve (Figure 8B) and tumor weight (Figure 8C). Treatment with exo-miR-552-5p led to an increase in the number of Ki67-positive tumor cells. Treatment with exo-Lv-inhibitor led to the opposite results (Figure 8D).

Finally, western blot analysis revealed higher levels of PTEN and TOB1 in the groups treated with exo-miR-552-5p. Conversely, lower levels of PTEN and TOB1 were seen with a decrease of miR-552-5p in exosomes (Figure 8E). These results suggest that exosomal miR-552-5p contributes to accelerating tumor progression by negatively regulating the PTEN-TOB1 axis.

## Discussion

Increasing evidence supports that exosomes play a crucial role in the proliferation, metastasis, angiogenesis, immune escape and drug resistance in GC 23. Exosomes, as a new type of cellular transmitter of information via proteins and various RNAs such as miRNAs, long non-coding (lncRNAs), and circular RNAs (circRNAs) in the tumor microenvironment, mediate malignant characteristics and pre-metastatic niche formation by regulating cell signaling 24-25. MicroRNAs have become attractive candidates as novel biomarkers for evaluating human disease development, particularly in the cancer field, and they are more stable in plasma if they are encapsulated by exosomes 26. Exosome secretion is reported to be significantly higher in tumor tissues than in non-cancerous tissues, and various tumor cells can contribute to the release of carcinogenic exosomal cargo 27-29. However, the potential mechanism of tumor-derived exosomal miRNAs has remained unclear, highlighting a critical and promising research direction.

In the current study, we found miR-552 to be significantly upregulated in both GES23739 and TCGA databases. Our studies demonstrate that miR-552-5p is highly expressed in GC tissues, plasma and cells, and this upregulation is negatively associated with prognosis. Functional studies revealed that miR-552-5p enhances a malignant phenotype by promoting cellular proliferation and migration while inhibiting apoptosis. Additionally, miR-552-5p stimulates the growth of xenografts and facilitates the formation of lung metastases. As previously reported, the exosomes released by cancer cells mediate communication to promote distant metastasis of primary tumor cells 30. Therefore, we detected levels of miR-552-5p in the circulatory system, including the plasma and plasma exosomes, and found that miR-552-5p is significantly enriched in exosomes of GC patient plasma compared to healthy volunteers, and overexpression of miR-552-5p in exosomes stimulates exosomal secretion. We separately co-cultured AGS and GES-1 cells with exosomes derived from SGC-7901 cells, followed by a series of functional experiments. The results indicated miR-552-5p can be stably packaged in exosomes, and exosomal miR-552-5p accelerates AGS and GES-1 cellular proliferation, migration, and EMT, and inhibits apoptosis, to accelerate tumorigenesis *in vivo*. Thus, miR-552-5p might function as an important carcinogenic factor in GC development.

We further explored the regulatory mechanism of miR-552-5p, and a bioinformatics analysis identified TOB1 as a regulatory target. TOB1 is a member of the TOB/B cell translocation gene (BTG) family that negatively regulates the receptor tyrosine kinase ERBB2, and it is thought to be a tumor suppressor 17. Overexpression of TOB1 enhances the apoptosis rate of breast cancer cells by regulation of the JNK and p38 pathways, and it enhances cell cycle arrest and radiosensitivity 31. TOB1 is negatively regulated by miR-32-5p to regulate radiosensitivity and inhibit the metastasis and invasion of colorectal cancer cells 32. In addition, TOB1 is regarded as a tumor suppressor in GC, attenuating the malignant phenotype of GC cells and inhibiting cell cycle progression and proliferation activity. However, phosphorylation of TOB1 at T172 and S320 can alter its antiproliferative activity in GC cells 33. Our luciferase assays indicated that instead of downregulating TOB1 directly, miR-552-5p binds the 3' UTR of PTEN to downregulate PTEN, leading in turn to downregulation of TOB1. PTEN is known to interact with TOB1 in lung and breast cancer 17, 34-35, and our immunoprecipitation results confirmed this interaction in SGC-7901 and AGS cells. PTEN stimulates the PI3K/AKT signaling pathway 36-37, and inactivation of PTEN can significantly inhibit tumor growth and sensitivity to immunotherapy 38-39. PTEN silencing is important for the suppression of the apoptosis regulator Bcl-2 family members and the activation of anti-apoptotic members 40.

The EMT is an essential step for the development of distant metastases. E-cadherin serves as an effective inhibitor of the invasion and growth of epithelial cancer types, while expression of N-cadherin and vimentin correlates with increased EMT and metastasis 41-42. PTEN loss or downregulation has been shown to induce the EMT 43. Dysregulated miRNAs play a specific role in tumor proliferation, metastasis, angiogenesis and drug resistance through post-transcriptional regulation of target agents, including PTEN/PI3K/Akt 44. In our study, both PTEN and TOB1 were expressed at high levels in adjacent non-cancerous tissue, while miR-552-5p was expressed at lower levels. In GC tissues, in contrast, the upregulation of miR-552-5p downregulated PTEN and inhibited its expression, leading in turn to downregulation of TOB1. This likely helps drive tumor proliferation, migration, and EMT as well as suppress apoptosis. Indeed, this is what we observed in recipient cells that took up miR-552-5p-carrying exosomes.

Collectively, our results support that miR-552-5p can promote the development of GC, in part through modulation of the PTEN/TOB1 pathway, and that exosomes support miR-552-5p-induced distant metastasis by functioning as vectors. However, whether the effect of exosomal miR-552-5p on distant metastasis is related to its effect on vascular endothelial cells and thus on angiogenesis remains to be elucidated in the future.

## Conclusion

We propose a model where miR-552-5p directly binds to the 3′ UTR of PTEN and then downregulates its interacting protein TOB1. This effect promotes tumor proliferation, migration, and EMT, and suppresses apoptosis. These effects can be reversed by raising the levels of PTEN. In addition, exosomal miR-552-5p can be internalized by recipient cells to promote malignant transformation at distant sites. Therapeutic targeting of the PTEN/TOB1 axis may therefore be an effective strategy in the treatment of GC.

## Figures and Tables

**Table 1:** Association of miR-552-5p level in GC tissues with clinical features

Clinical features	Group	miR-552-5p Low expression	miR-552-5p High expression	P vale
Sex	Female	5	7	0.465
	Male	11	9	
Age	≤56	8	7	0.723
	>56	8	9	
T	T1-T3	11	3	0.004^**^
	T4	5	13	
N	N0-N1	14	4	<0.001^***^
	N2-N3	2	12	
Tumor stage	I/II	13	1	<0.001^***^
	III/IV	3	15	

T, tumor; N, node. **, P<0.01; ***, P<0.001.

**Table 2:** The correlations of the plasma exosomal miR-552-5p level with GC clinical features

Clinical features	Group	Exo-miR-552-5p Low expression	Exo-miR-552-5p High expression	P vale
Sex	Female	6	8	0.464
	Male	9	7	
Age	≤56	6	7	0.713
	>56	9	8	
T	T1-T2	10	2	0.003^**^
	T3-T4	5	13	
N	Negative	11	4	0.011^*^
	Positive	4	11	
Tumor stage	I/II	10	3	0.01^*^
	III/IV	5	12	

T, tumor; N, node. *, P<0.05; **, P<0.01.

## Abbreviations

GC: Gastric cancer
EMT: epithelial-mesenchymal transition
TEM: Transmission electron microscopy
CCK‑8: Cell counting kit‑8
EdU: 5-ethynyl-2'-deoxyuridine
CO-IP: Co-immunoprecipitation
GEO: Gene Expression Omnibus
TCGA: The Cancer Genome Atlas
TOB1: Transducer of ERBB2.1
